# CRISPR/Cas Derivatives as Novel Gene Modulating Tools: Possibilities and In Vivo Applications

**DOI:** 10.3390/ijms21093038

**Published:** 2020-04-25

**Authors:** Xingbo Xu, Melanie S. Hulshoff, Xiaoying Tan, Michael Zeisberg, Elisabeth M. Zeisberg

**Affiliations:** 1 Department of Cardiology and Pneumology, University Medical Center Göttingen, Robert-Koch-Str. 40, 37075 Göttingen, Germany; xingbo.xu@med.uni-goettingen.de (X.X.); melanie.hulshoff@med.uni-goettingen.de (M.S.H.); 2 German Centre for Cardiovascular Research (DZHK), 37075 Göttingen, Germany; xiaoying.tan@med.uni-goettingen.de (X.T.); mzeisberg@med.uni-goettingen.de (M.Z.); 3 Department of Pathology and Medical Biology, University Medical Center Groningen, Hanzeplein 1, 9713 GZ Groningen, The Netherlands; 4 Department of Nephrology and Rheumatology, University Medical Center of Göttingen, Robert-Koch-Str. 40, 37075 Göttingen, Germany

**Keywords:** CRISPR/Cas, genome editing, transcriptional regulation, dCas9 derivatives

## Abstract

The field of genome editing started with the discovery of meganucleases (e.g., the LAGLIDADG family of homing endonucleases) in yeast. After the discovery of transcription activator-like effector nucleases and zinc finger nucleases, the recently discovered clustered regularly interspaced short palindromic repeats (CRISPR)/CRISPR associated proteins (Cas) system has opened a new window of applications in the field of gene editing. Here, we review different Cas proteins and their corresponding features including advantages and disadvantages, and we provide an overview of the different endonuclease-deficient Cas protein (dCas) derivatives. These dCas derivatives consist of an endonuclease-deficient Cas9 which can be fused to different effector domains to perform distinct in vitro applications such as tracking, transcriptional activation and repression, as well as base editing. Finally, we review the in vivo applications of these dCas derivatives and discuss their potential to perform gene activation and repression in vivo, as well as their potential future use in human therapy.

## 1. Introduction

In the last ten years, major breakthroughs have been made in the field of gene editing, which is the process where DNA is modified, deleted, inserted or replaced. The most recent discovery of the clustered regularly interspaced short palindromic repeats (CRISPR)/CRISPR associated proteins (Cas) system has opened up many novel opportunities as well as applications for gene editing both in vitro and in vivo. In this review, we provide an overview of the different gene editing techniques including the CRISPR/Cas system, which was discovered in 2012. After this finding, different Cas proteins were developed with unique features that allow for distinct gene editing approaches. Here, we summarize these different Cas proteins and detail their specific features. Besides the development of different Cas proteins, endonuclease-deficient Cas proteins (dCas) allow for additional applications of the CRISPR/Cas system. These endonuclease-deficient Cas proteins (dCas) can be fused to effector domains which exert additional functions such as transcriptional activation or repression, but also tracking and base-editing. These dCas effectors as well as their functions and in vivo applications will be the main focus of this perspective.

## 2. Gene Editing Techniques

**Figure 1:**
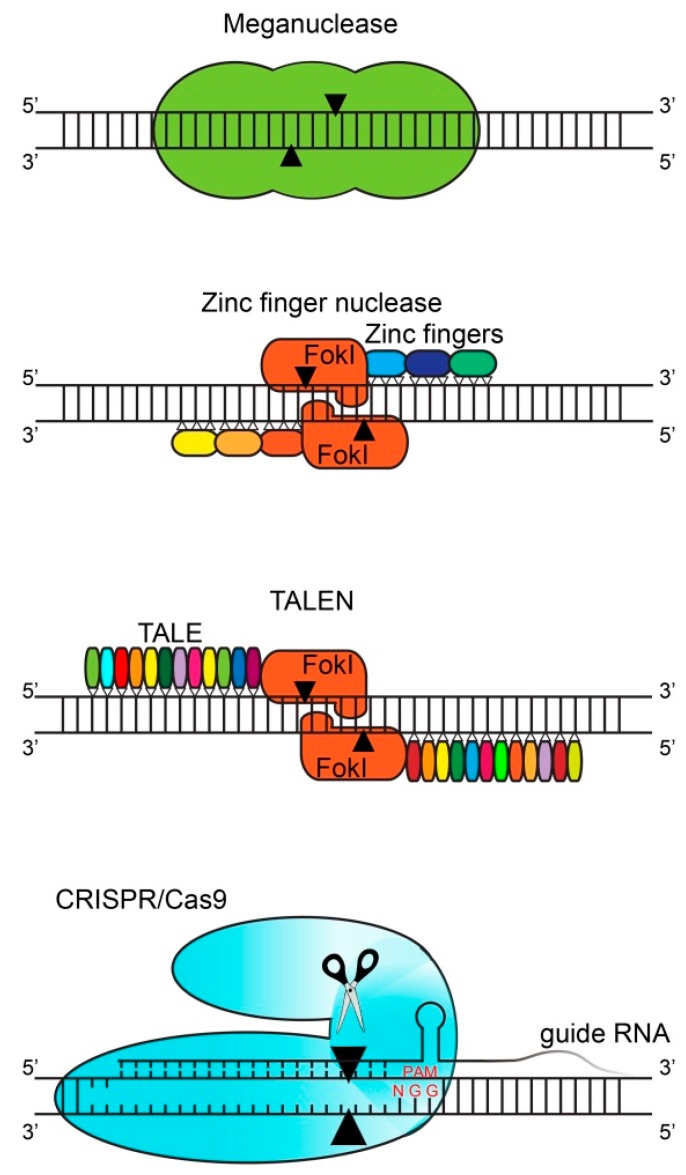
Gene editing techniques. Overview of the different gene editing designer nucleases. This figure depicts the initially discovered meganucleases. In addition, the zinc finger and transcription activator-like effectors (TALE) are depicted, which are fused to Fokl nuclease monomers which cleave the DNA upon dimerization. Each DNA-binding module is indicated with a different color. The most recently discovered RNA-guided clustered regularly interspaced short palindromic repeats (CRISPR)/CRISPR associated proteins-9 (Cas9) system is illustrated as well. Black arrows represent the cleavage site.

Gene editing started with the discovery of meganucleases in yeast as well as in other small organisms. There are hundreds of meganucleases which each contain a recognition sequence (homing site) between 14 and 40 base pairs (Figure 1) [1]. This large recognition site ensures great locus specificity together with low off-target effects. At the same time, the large recognition site is also the main limitation of meganucleases since their recognition site only occurs once (or sometimes not at all) in the genome of a given organism. This limitation can be circumvented by modifying the recognition site of the particular meganuclease [2], which is time-consuming and labor-intensive.

In 1994, the zinc finger nucleases (ZFN) were discovered as the first modulated gene editing tools [3]. ZFN consist of nucleases which are coupled to certain zinc fingers which each recognize triplets of base pairs (codons; Figure 1) [4]. To increase the binding specificity, several zinc fingers are attached together (usually three) to reach a binding site of nine nucleotides (three codons). The same holds for the complementary strand, so in total an 18-nucleotide specificity is reached. These zinc fingers can be engineered in a specific way to recognize the DNA region of interest. These connected zinc fingers are then fused to cleavage domains of endonucleases (usually to Fokl nuclease monomers) [5]. The sequence in between the two ZFN binding sites is the so-called spacer region, consisting of 5–7 nucleotides where Fokl monomers form a catalytically active dimer, which cleaves the DNA, resulting in double-strand breaks [6]. The production of an efficient ZFN might require multiple rounds of re-designing and optimizations (because each of the selected ZF might affect the binding affinity of the adjacent ZFs), which is labor-intensive, time-consuming and most importantly expensive.

In 2009, the transcription activator-like effector nucleases (TALEN) were developed, which function in a similar way but have increased specificity when compared with ZFN [7,8]. Transcription activator-like effectors (TALE) were discovered in bacteria and are able to bind to single nucleotides (instead of triplets of nucleotides by zinc fingers) [9]. Similar to ZFN, several TALE are attached together (i.e., 12–31 repeats per TALEN) to target the DNA sequence of interest and are fused to the Fokl nuclease monomers (Figure 1) [10]. Similarly, the second TALEN monomer is designed for the complementary strand. These Fokl nuclease monomers will again form a catalytically active dimer in the spacer region and cleave the DNA. The procedure of producing new ZFN and TALEN is labor-intensive since for every DNA target region of interest, novel ZFN and TALEN have to be generated.

In 2012, the most recent gene editing tool was discovered: clustered regularly interspaced short palindromic repeats (CRISPR) and CRISPR associated genes (Cas) [11]. The CRISPR genomic locus consists of Cas genes (which code, e.g., for the nuclease Cas9) and a leader sequence followed by repeat sequences which are flanked by spacer regions. Originally, CRISPR was discovered in bacteria and contributes to the immune defense system where foreign DNA from viruses or fungi is inserted within the repeat sequences of the CRISPR genomic locus [12,13]. These repeat sequences are subsequently transcribed into so-called CRISPR RNAs (crRNAs) which recognize foreign DNA and recruit a Cas protein to cleave the foreign DNA. This system can be adapted by the design of a so-called single guide RNA (sgRNA), which is a custom-designed short crRNAs sequence fused to the scaffold trans-activating crRNA sequence (tracrRNA) and guides the Cas9 nuclease to a specific DNA region of interest. Another essential element for the Cas nuclease is the protospacer adjacent motif (PAM) which is a short specific sequence following the target DNA sequence that is needed for Cas nuclease-mediated cleavage [11]. The precise location of the Cas nuclease can be adapted by changing the sgRNA to a specific locus. This sgRNA molecule is cheaper and easier to generate compared to the production of ZFN and TALEN, which is an advantage of the CRISPR/Cas system. The limitation of CRISPR, but also of ZFN and TALEN, is the presence of off-target effects. These off-target effects have been reduced by the discovery of the so-called high-fidelity CRISPR/Cas system, where four amino acids have been substituted in *Streptococcus pyogenes* Cas9 (SpCas9) by Kleinstiver and his colleagues. These four amino acid substitutions alter the energetics of the binding of SpCas9 to the DNA, and thereby the specificity, which diminished off-target effects [14]. A comparison of the different gene editing techniques is provided in Table 1.

## 3. Variety of Cas Proteins

The most commonly used Cas9 protein is derived from *Streptococcus pyogenes* (SpCas9) [11]. The use of SpCas9 is limited by its 5′-NGG-3′ (where N = G, C, T or A) PAM motif, which constricts the range of targetable sites in the DNA [15]. The wild-type SpCas9 is, therefore, engineered to gain different PAM specificities, which enable the targeting of previously inaccessible sites. These engineered SpCas9 mutants include VQR (D1135V/R1335Q/T1337R) with 5′-NGAN-3′ as the PAM, EQR (D1135E/R1335Q/T1337R) with 5′-NGAG-3′ as the PAM and VRER (D1135V/G1218R/R1335E/T1337R) with 5′-NGCG-3′ as the PAM (Table 2) [15]. SpCas9 consists of 1368 amino acids, which restricts its use in adeno-associated virus (AAV) vector-based therapeutic applications (which will be discussed later in this review). More than 1kb shorter than SpCas9 are the Cas9 proteins derived from *Staphylococcus aureus* (SaCas9), *Neisseria meningitidis* (NmCas9), *Streptococcus thermophilus* (St1Cas9) and *Brevibacillus laterosporus* (BlatCas9; Table 2) [16,17,18,19]. The targeting range of SaCas9 is also increased by a modified PAM recognition site (from 5′-NNGRRT-3′ to 5′-NNNRRT-3′) [19,20]. NmCas9 recognizes a 5′-NNNNGATT-3′ PAM, whereas St1Cas9 and BlatCas9 recognize 5′-NNAGAAW-3′ and 5′-NNNNCNDD-3′ (where D = A, G or T), respectively [16,17,18]. SaCas9, NmCas9 and St1Cas9 all have a strict PAM [16,17,19,20], whereas the PAM of BlatCas9 is less restrictive since it has a strong preference for just a single nucleotide [18]. Cas9 derived from *Francisella novicida* (FnCas9) also has a less restrictive PAM but with 1629 amino acids, it is significantly larger than the other Cas9 orthologs [21]. FnCas9 consists of a wild-type and a mutated form which recognize 5′-NGG-3′ and 5′-YG-3′ (where Y = C or T), respectively (Table 2) [21]. Even though BlatCas9 and FnCas9 have a less restrictive PAM, there are less application examples when compared to the other Cas9 orthologs (most likely because they were discovered later).

Besides the different Cas9 orthologs, other Cas proteins have been discovered as well including Cpf1 (CRISPR from *Prevotella* and *Francisella* 1 also known as Cas12), Cas13 and Cas14 [22]. Cpf1 has various unique characteristics: (1) it is a single RNA-guided endonuclease which lacks tracrRNA, (2) it has a strict T-rich PAM (5′-TTTN-3′) when compared to the more G-rich PAM of Cas9 proteins, and (3) where Cas9 facilitates blunt ends, Cpf1 facilitates sticky ends with a 4 or 5 nucleotide 5′ overhang [22]. To date, there are several known Cpf1 orthologs with robust nuclease activity: *Acidaminococcus sp.* Cas12 (AsCpf1), *Lachnospiraceae bacterium* (LbCpf1) and *Bacillus hisashii* Cas12b (BhCas12b; Table 2) [22,23]. In addition, Cas12c, Cas12g, Cas12h and Cas12i have been characterized which demonstrate RNA-guided dsDNA interference activity [24].

In addition to Cas9 and Cpf1, CasX enzymes represent a distinct family of RNA-guided genome editors. CasX enzymes use unique structures for programmable double-stranded DNA binding and cleavage [25].

Where Cas9 and Cpf1 are widely used to induce DNA breaks (as well as the recently discovered CasX enzymes), Cas13a (previously called C2c2) cleaves single-stranded RNA [25,26,27]. Similar to Cpf1, Cas13a lacks tracrRNA and is guided by a single crRNA [27]. RNA cleavage is mediated by catalytic residues in the two conserved higher eukaryotes and prokaryotes nucleotide-binding (HEPN) domains and is dependent on a 3′ H (non-G) protospacer flanking site (PFS) following the RNA target site [27]. The first characterized Cas13 protein derived from the bacterium *Leptotrichia shahii* (LshCas13a) [27]. The later characterized *Leptotrichia wadei* Cas13a (LwaCas13a) exhibits a higher cleavage activity than LshCas13a and does not require a PFS (Table 2) [26]. After the discovery of LwaCas13a, researchers identified *Prevotella sp. P5-125* Cas13b (PspCas13b), which also does not require a PFS (Table 2) [28]. PspCas13b-mediated mRNA knockdown is more efficient when compared to LwaCas13a [28].

The most recently discovered Cas13 protein is *Ruminococcus flavefaciens* Cas13d (RfxCas13d) and is also not dependent on a PFS (Table 2) [29]. This Cas13 protein has the smallest protein size which allows packaging into AAV vectors, and thus enables AAV vector-based therapeutic applications (will be discussed later in this review) [29]. In summary, the main differences between Cas9 and Cas13 include the PAM, the length of the sgRNA and the cleavage of RNA vs. DNA.

Cas9 and Cas13 can both serve as gene silencing tools for protein coding genes. The Cas9 system utilizes non-homologous end joining-based DNA damage repair to create a DNA insertion/deletion (indel) mutation to generate a frame shift or a point mutation (e.g., premature stop codon, non-functional protein) [11]. In this way, a premature stop codon is very likely to form and a functional protein will not be translated. However, for non-protein coding genes such as long non-coding RNAs, utilizing Cas9 is unlikely to show gene silencing effects, whereas Cas13 can target and cleave RNA, thereby triggering the RNA degradation pathway [26,28]. In addition to programmable RNase activity, Cas13 exhibits collateral activity after recognition and cleavage of a target transcript, leading to non-specific degradation of any nearby transcripts regardless of complementarity to the spacer [30,31]. This property enables Cas13 to be well suited for nucleic acid detection. When the enzyme recognizes its target in vitro, it becomes activated and promiscuously cleaves RNA species in the solution. Cas13-based detection is specific and can be tuned for single-nucleotide distinction at any position on the target. This nucleic acid detection technology is called specific high-sensitivity enzymatic reporter unLOCKing (SHERLOCK) [30,31].

The most recently discovered Cas14 family consists of the smallest RNA-guided nucleases, which depend on a tracRNA and, in contrast to the other Cas proteins, targets single-stranded DNA (Table 2) [32]. The cleavage of single-stranded DNA by Cas14 is not dependent on restrictive sequence requirements (PAM) [32]. Altogether, these different Cas proteins enable a wide range of targeting sites and applications to perform CRISPR/Cas-mediated gene editing at either the DNA or RNA level (Table 2).

## 4. dCas Derivatives and their Functions

The CRISPR/Cas system can be repurposed in several ways by modification into an endonuclease-deficient Cas protein (dCas). dCas9 is generated via the introduction of two mutations in the cleavage domains of Cas9 (D10A and H840A). dCas9 is still capable to bind to the DNA in a sgRNA-mediated manner but does no longer cleave the DNA. Different effector domains with distinct gene regulatory functions, such as transcriptional activators, repressors or base editing domains, can be fused to dCas9. The subsequent introduction into cells results in sgRNA-guided gene activation, gene repression or base editing of the targeted locus. dCas9 can also be used to perform fluorescent imaging. These Cas9 derivatives will be discussed in more detail below.

### 4.1. Chromatin Visualization and Fluorescent Imaging with dCas Derivatives

**Figure 2:**
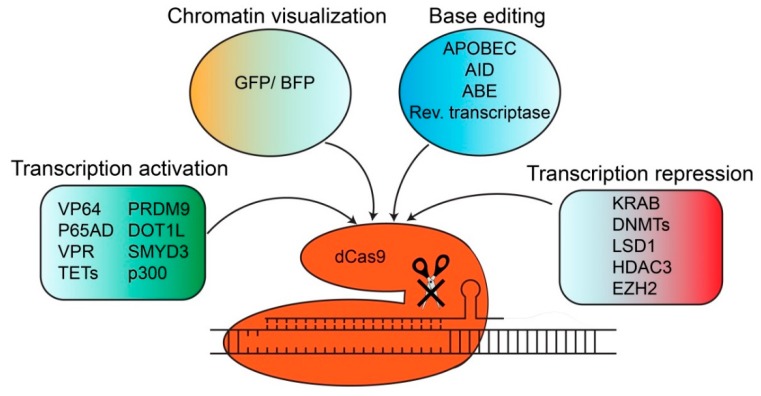
Endonuclease-deficient Cas9 proteins (dCas9) derivatives and their purpose. Overview of the different dCas9 derivatives. Their purpose includes transcriptional activation (via transcriptional activators or epigenetic modifiers), chromatin visualization, base editing and transcriptional repression (via repressor domains or epigenetic modifiers). Rev. transcriptase: reverse transcriptase.

One possibility is to use an sgRNA-dependent enhanced green fluorescent protein (EGFP)-tagged dCas9 protein to dynamically image repetitive elements in both telomeres and coding genes (Figure 2) [33]. This system is composed of three components: cells stably expressing a doxycycline-inducible EGFP-tagged dCas9 and Tet-On 3G/BFP transactivator which are transduced with lentiviral vectors expressing the respective sgRNAs [33]. Non-repetitive sequences can be imaged as well by the design of different sgRNAs at multiple adjacent sites of the locus of interest [33]. This strategy was used to visualize a non-repetitive region of the *MUC4* locus [33]. Importantly, the Cas9 fusion protein did not disrupt telomere dynamics. Genomic labeling by dCas9-EGFP is not destructive and also allows to observe native chromatin dynamics. The authors designed sgRNAs targeting exon 2 and intron 3 of the *MUC4* gene in HeLa cells to investigate the chromosome reorganization dynamics during cell replication. Therefore, dCas9 fluorescent imaging represents a new approach to study chromatin conformation and dynamics in both short time frames and long-term processes such as mitosis [33]. Another possibility for fluorescent imaging is the so-called dCas9-SunTag system. This is a protein tagging system for signal amplification in fluorescent imaging [34]. This system consists of a protein scaffold, which is a repeating peptide array called SunTag (10xGCN4), which can recruit multiple copies of an antibody (single-chain variable fragment [scFV]-fused protein such as GFP) [34]. This system can be used for fluorescent imaging, where SunTag can recruit up to 24 copies of GFP which results in the long-term imaging of single protein molecules in living cells [34]. This system can also be used to recruit multiple copies of effector domains responsible for transcriptional activation, which will be discussed in more detail below.

### 4.2. Transcriptional Activation with dCas9 Derivatives

Recruitment of transcriptional factors or activation domains to initiate gene expression can be achieved by fusion of various activation domains to dCas9 (Figure 2). dCas9-based gene reactivation methods which upregulate the expression of endogenous genes have the advantage over gene over-expression techniques since 95% of the human genes are alternatively spliced, and forced expression of any single mRNA transcript would distort the original homeostatic ratios of the endogenous transcripts [35]. Examples of these activation domains are the C-terminal of the VP64 acidic transactivation domain [36,37] or the p65 activation domain (p65AD) [36]. To enhance the transcriptional activity, these transcriptional activators can also be combined into a tripartite activator called VPR (which is a combination of VP64, p65 and Rta) [38]. These activation domains are capable to activate both endogenous coding and non-coding genes and can target several genes at the same time by using multiple sgRNAs [38]. The previously mentioned dCas9-SunTag system is also used to recruit multiple copies of VP64 to dCas9-VP64, which enhances the transcriptional activation by dCas9-VP64 [34]. Since these methods are based on the over-recruitment of transcription factors, this often leads to gene expression levels much higher than physiological levels which largely limit the in vivo applications.

Besides fusing transcriptional activators to dCas9, it is also possible to fuse dCas9 to epigenetic-modifying domains. Here, the gene is activated via their endogenous transcription system and is therefore expressed at physiological levels [39,40]. In general, gene regulation via dCas9-based epigenetic effectors includes DNA promoter methylation and histone modifications. DNA promoter methylation is responsible for gene silencing, whereas histone modifications induce either gene activation (via histone acetylation or trimethylation of histone H3 lysine 4) or gene silencing (e.g., dimethylation of histone H3 lysine 4). Examples of epigenetic-modifying domains responsible for gene activation are the catalytic domain of ten-eleven translocation methylcytosine dioxygenases (TETs) or the histone acetyltransferase p300. TETs are responsible for reversing DNA promoter methylation via hydroxymethylation, thereby re-activating gene expression. We and others have shown that fusion of dCas9 to the catalytic domain of TET1 [41,42,43] and TET3 [44] enables gene-specific demethylation which results in the re-activation of gene expression [41,42,43,44]. The dCas9-SunTag system is also used to recruit multiple copies of the catalytic domain of TET1 to dCas9-TET1CD to perform targeted DNA demethylation [42]. Another strategy includes the recruitment of multiple copies of the catalytic domain of TET1 by using an MS2 coat protein [41]. Another possibility of epigenetic editing is the fusion of dCas9 to the catalytic core of the histone acetyltransferase p300 [40]. P300 acetylates histone H3 lysine 27, which induces the transcriptional activation of the target genes from the promoters and both the proximal and distal enhancers [40]. dCas9 can also be fused to the histone methyltransferase SMYD3 (SET and MYND domain containing protein 3) to facilitate the trimethylation of histone H3 lysine 4 (H3K4me3), which results in the transcriptional activation of silenced target genes [45]. The catalytic core of the histone methyltransferase PRDM9 also induces H3K4me3 marks when fused to dCas9, which results in transcriptional activation [46]. The H3K79 methyltransferase DOT1L also enables re-expression of genes when fused to dCas9 [46]. The mix of both PRDM9 and DOT1L enabled the stable induction of gene re-expression [46]. Interestingly, the presence of DNA methylation hampers gene re-expression induced by both PRDM9 and DOT1L [46]. This raises the possibility to combine DNA demethylation effector domains with histone-modifying effector domains to enhance the efficacy of dCas9-based transcriptional activation. Altogether, this demonstrates that both VP64-based artificial transcription factors as well as epigenetic effectors can be fused to dCas9 to perform gene activation.

dCas9-based gene activation systems (by fusing dCas9 to different activators such as VP64, p65, Rta or HSF1) showed great gene reactivation effects, but they mainly depend on using a pool of different guide RNAs, which makes it more difficult to use them effectively in genome-wide screens. Additionally, these ectopic transcription factors increase the gene reactivation level beyond physiological conditions, which is likely to bring unwanted side effects. Also, VP64 and Rta are derived from the herpes simplex viral protein 16 and Epstein–Barr virus Rta Protein, respectively, which might lead to cell type-specific efficiency and toxicity issues [47].

TET protein-mediated DNA demethylation and histone modification enzyme-induced chromatin remodeling can also reactivate gene expression. These methods rely on increasing the chromatin accessibility of the gene promoter/enhancer regions to recruit endogenous transcription factors, thereby enhancing the transcription. These methods have the advantage over dCas9 fusion with activators such as VP64, p65, Rta or HSF1 since they maintain the reactivated gene expression within a physiological level [39,40]. However, dCas9-TET fusion-mediated gene reactivation techniques can only reactivate those genes which are silenced by promoter hypermethylation, and it is also important to know the critical CpG sites, whose methylation controls the gene expression, in order to design effective guide RNAs to reactivate gene expression [44]. Moreover, dCas9-derived histone modifiers usually need a pool of different guide RNAs in order to successfully restore gene expression [40].

### 4.3. Transcriptional Repression with dCas9 Derivatives

Besides transcriptional activation, effector domains which facilitate transcriptional repression can also be fused to dCas9 (Figure 2). Examples of these repressor domains are the KRAB (Kruppel-associated box) domains of Kox1 [36,48]. Also here, epigenetic modifiers can be used as well to perform sgRNA-guided dCas9-based transcriptional repression. These include the catalytic domain of DNA methyltransferases (DNMTs) such as DNMT3A and DNMT3L, which induce site-specific DNA promoter methylation, which results in gene silencing and is heritable across mitotic division [48,49,50]. Methylation of a larger part of the promoter can be achieved via the design of multiple sgRNAs at adjacent sites [49]. In addition, the combination of several repressor domains such as KRAB, DNMT3A and DNMT3L allows stable and stimulation-resistant gene silencing [48]. Besides inducing DNA promoter methylation, histone-modifying enzymes can also be used to induce dCas9-based transcriptional repression. The histone demethylases LSD1 and KDM1A can be fused to dCas9 to allow transcriptional silencing [51]. LSD1 demethylates H3K4me2 histone modifications that correlate with active enhancers, which results in the loss of H3K4me2 as well as H3K27Ac (which is indicative of active enhancers) [51]. LSD1 can therefore be used to identify which enhancers are critical for the appropriate expression of genes in, for example, the stem cell state [51]. Also, the histone methyltransferase EZH2 can be fused to dCas9 to facilitate gene silencing [52]. Besides histone demethylases/methyltransferases, histone deacetylases can also be fused to dCas9 to induce transcriptional repression. An example of this is the histone deacetylase 3 (HDAC3), which when fused to dCas9, can induce locus-specific histone deacetylation that results in gene silencing [53]. Here, the location of the sgRNA is critical for the dCas9-HDAC3 activity: only the sgRNAs located adjacent to the H3K27ac marks promote a dCas9-HDAC3-dependent effect on the transcription [53]. This shows that both repressor domains such as KRAB as well as epigenetic-modifying enzymes such as DNMTs and histone demethylases and deacetylases can be fused to dCas9 to induce sgRNA-guided gene silencing of the desired locus. It is important to note that different combinations of histone demethylases/methyltransferases and DNA methyltransferases are required to achieve maximal repression at different loci (e.g., the requirement of histone methyltransferases can be locus specific) [54].

The advantage of dCas9-based gene repression modules (fused with KRAB, LSD1, KDM1A or HDAC) is that it can efficiently and specifically decrease gene expression by up to 99% [55]. As compared to the conventional RNA interference (RNAi) method, dCas9-based gene silencing methods are easy to modify and can be used in ex vivo cell therapy as well [56]. This repression modules-mediated silencing of the target gene expression is facilitated by the histone modifications of the cell itself. This means that long-term suppression of gene expression results in additional DNA methylation marks, which leads to an enhanced robustness of the gene suppression [48,57].

Notably, dCas9-fused DNMTs also hold a great ability in gene silencing, however, this is only applicable for those genes which have CpG-rich regions in the promoter (half of all human genes contain a CpG-rich region). In addition, Galonska et al. have reported that dCas9-DNMT modules have global off-target effects by adding methylation footprints that are independent of the sgRNA and delivery methods [58].

### 4.4. Base Editing with dCas9 Derivatives

The CRISPR/Cas9 system can also be repurposed to perform base editing in the DNA to correct mutations relevant to human disease (Figure 2). This enables the direct and irreversible conversion of a target base in the DNA into another one. Notably, Cas9-dependent base editing functions in a programmable manner (sgRNA-guided) and no longer requires double-stranded DNA cleavage or a donor template. The fusion of different effector domains to dCas9 enables base editing of different bases. These effector domains include single-strand-specific deaminases that convert cytosine (C) into uracil (U), which pairs with adenine during replication, resulting in a C to T conversion. The cytidine deaminase enzyme APOBEC1 enables C to T substitution [59,60,61], whereas the activation-induced cytidine deaminase (AID) enables C to T/G substitution [62,63]. An additional uracil glycosylase inhibitor (UGI) is added to the dCas9-cytidine deaminase fusion protein to prevent excision of uracil (and thus uracil base excision repair), and thereby increases the base editing efficiency and product purity. Nickase activity of the Cas9(D10A) protein manipulates cellular mismatch repair into replacing the G in the non-edited strand [63]. These components can also be delivered as an all-in-one ribonucleoprotein (RNP) complex to establish DNA-free base editing [61]. Another possibility is to mutate cytidine deaminase domains to narrow the width of the base editing window from approximately 5 nucleotides to either 1 or 2 nucleotides [59]. This results in increased specificity since it excludes the neighboring cytosines in the 5 nucleotide-window which would otherwise be base-edited as well. The addition of dCas9 to AID can be achieved via either direct fusion or by attaching an SH3 (Src 3 homology) domain to the C terminus of dCas9 and an SHL (SH3 interaction ligand) to the C terminus of AID [62]. The additional blockage of uracil N-glycosylase (UNG), which is also responsible for uracil cleavage, and thus initiation of uracil base excision repair, was used to develop the fourth generation of base editors (BE4 (*S. pyogenes* Cas9-derived base editor) and SaBE4 (*Staphylococcus aureus* Cas9-derived BE4)) [63]. Furthermore, fusion of these fourth generation of base editors to the bacteriophage Mu dsDNA end-binding protein Gam reduced the formation of undesired base editing-induced indels and increased the product purity [63]. Another effector domain that can be fused to dCas9 are the so-called adenine base editors (ABE), which facilitate A to G substitution in the DNA [64]. Here, dCas9 is fused to tRNA adenosine deaminase which performs efficient and high-purity base editing while generating a low rate of indels [64]. It is important to note that DNA base editing induces off-target RNA mutations, which can be eliminated by engineering deaminases (e.g., cytosine base editors (CBE) or ABE variants) [65]. Altogether, this demonstrates the multiple applications of Cas9 derivatives including fluorescent imaging, transcriptional activation or repression, and base editing.

### 4.5. Prime Editing

Base editing ingeniously avoids DNA breaks and was thought to minimize inaccuracy [66]. However, this strategy only offers limited options as it can only make 4 out of the 12 possible base pair changes and some recent works have suggested it is not as precise as scientists first thought [67]. Recently, David R. Liu’s group discovered a new type of gene editing, which is called prime editing. Prime editing steers around shortcomings of Cas9 and base editing by modifying both the Cas9 protein and the gRNA. Here, the nickase Cas9(H840A) cleaves only one strand of DNA, leaving the other intact. An engineered prime editing guide RNA (pegRNA) contains an RNA template for a new DNA sequence to be incorporated into the cleaved strand of DNA at the target location. In addition, a reverse transcriptase enzyme which is fused to the Cas9 protein enables to synthesize a new DNA strand from the RNA template to be inserted at the cleaved site. Prime editing is considered a promising novel part of the CRISPR tool kit [66]. Nevertheless, its robustness needs to be established in different in vitro models as well as in vivo.

### 4.6. Functions of Cas13 Derivatives

Where Cas9 derivatives can be used to induce transcriptional activation or repression at the DNA level, Cas13 derivatives can be used for RNA targeting. Similar to dCas9, dCas13 is able to bind to the target RNA but is unable to cleave it. Further, and also similar to dCas9, an EGFP-tagged dCas13 can be used to track transcripts in living cells [26]. Moreover, dCas13a can also be fused to a KRAB domain to repress transcription [26]. In addition, dCas13 proteins can also be repurposed for adenine (A) to inosine (I) base editing [28,68]. Iosine can base pair with cytidine and can therefore be corrected to guanine, resulting in an A to G conversion. Base editing at the RNA level holds promise for treating genetic diseases, where disease-relevant sequences containing pathogenic mutations can be edited to yield functional proteins [28]. Both dCas13a and dCas13b can be fused to the deaminase domain of ADAR 2 (adenosine deaminase acting on RNA type 2) which enables direct A to I deaminase activity to transcripts in mammalian cells [28,68]. The ADAR2 deaminase domain can also be mutated (E488Q) to increase the A to I editing rates [28]. This system is called RNA editing for programmable A to I replacement (REPAIR). This system has recently evolved into RNA editing for specific C to U exchange (RESCUE), which is capable of both C to U and A to I editing [69,70]. Finally, Cas13-based derivatives are able to promote mRNA translation and mRNA decay [71]. The fusion of dCas13b to the two most well-characterized m^6^A (which is the most prevalent mRNA modification) reader proteins YTDHF1 and YTDHF2 results in enhanced translation and the induction of mRNA degradation, respectively [71]. Taken together, this demonstrates the versatile use of Cas derivatives to perform several cellular applications such as imaging, transcriptional modulation and base editing (Table 3).

## 5. In Vivo Applications of dCas Derivatives

The CRISPR/Cas derivatives have distinct effects in vitro, such as transcriptional activation or repression, but only some of them have been demonstrated to function in vivo. The first description of dCas9-mediated transcriptional activation in vivo was in drosophila [73]. Here, dCas9-VPR enabled the activation of endogenous genes in drosophila which induced dominant phenotypes [73,74]. dCas9-VPR was the most optimal for the transcriptional activator in drosophila and outperformed dCas9-VP64 and dCas9-SAM (synergistic activation mediator) [73,74]. dCas9-SAM includes dCas9-VP64 in combination with two MS2 protein-fused additional activator domains, p65 and HSF1 (heat shock factor 1), which are recruited to the sgRNA tail [74]. dCas9-VPR-based transcriptional activation in drosophila holds great potential for overexpression studies where a tissue-specific Gal4-driven dCas9-VPR can be crossed to flies expressing distinct sgRNAs, which is both cheap and scalable [73,74]. Transcriptional activation using dCas derivatives has also been shown in mice in several studies. Here, a neuron-optimized dual lentiviral dCas9-VPR system is used for robust and gene-specific regulation in rats [75]. Further, light-sensitive gene transcriptional activation was performed using dCas9-SAM which is delivered into the muscles of mice via electroporation [76]. In a different study, dCas9-VP64 was used to perform transcriptional activation in a cancer mouse model [56]. Interestingly, it was suggested that the targeting of dCas9-VP64 downstream of the transcriptional start site results in gene silencing instead of gene activation [56]. This might be due to dCas9-VP64 acting as a transcriptional roadblock by interfering with transcriptional initiation/elongation [56]. In another cancer mouse model, dCas9-SunTag (with recruiting VP64 domains) was used to induce transcriptional activation [77]. Altogether, CRISPR/dCas9 derivatives allow to study the impact of individual genes on disease phenotypes such as cancer in vivo. The fourth study uses CRISPR/dCas9 derivatives to perform simultaneous transcriptional activation of multiple genes and long non-coding RNAs, which are more than 200-nucleotides long, which belong to the non-coding RNA group of epigenetic modifiers in the mouse brain [78]. This study combines two CRISPR/dCas9 derivatives into one: it uses dCas9-SunTag but replaces VP64 with p65-HSF1 (which were previously used in dCas9-SAM) now called dCas9-SunTag-p65-HSF1 (SPH) [78]. The targeted activation of three endogenous neurogenic transcription factors resulted in the efficient conversion of astrocytes into functional neurons in vivo via AAV8 vectors [78]. A robust activation of multiple genes was also achieved in the liver using dCas9-SPH [78].

As described before, dCas9-based transcriptional activation can not only occur via fusion of dCas9 to the transcriptional activators but also to the epigenetic modifiers, such as TETs, which induce DNA demethylation, and thus the re-activation of gene expression. The dCas9-SunTag (with recruiting TET1CDs) is used to perform targeted demethylation, and thus the upregulation of genes in the brain of mouse fetuses via in utero electroporation [42]. dCas9-TET1 is also used in another study to facilitate demethylation in vivo in the brain and skin of mice via lentiviral vectors [79]. We have also demonstrated that dCas9-TET3CD induces gene-specific re-activation and demethylation, which results in the amelioration of kidney fibrosis in vivo via lentiviral vectors [44].

Besides transcriptional activation, CRISPR-dCas9-based transcriptional repression has also been reported in a few in vivo studies. In general, CRISPR-dCas9 derivatives are delivered in vivo via lentiviral vectors [44] and electroporation [80], but mostly AAV vectors [77,78,81,82], because of its low toxicity and immunogenicity. However, AAV vectors are associated with delivery challenges since the large size of CRISPR-dCas9 derivatives exceeds the packaging limit of AAV vectors. To circumvent the AAV vector packaging limitation, in vivo studies made use of Cre-dependent Cas9 activation (which also allows cell type-specific activation) [77,78], the so-called split-intein system [82], or a smaller Cas9 protein (SaCas9) [81]. The split-intein system enables the fusion of two separate parts of CRISPR-dCas9 derivatives. Here, the N-terminal of a CRISRP/Cas9 derivative is fused to the N-intein, whereas the C-terminal is fused to C-intein. Upon transduction, the two intein proteins recognize and excise themselves, resulting in a complete and functional dCas9 protein. This system was used for the transcriptional activator dCas9-VP64-RTA as well as the transcriptional repressor dCas9-KRAB and allowed in vivo gene activation and repression, respectively [82]. The utilization of additional activator domains (VP64 or p65) improved gene activation, whereas additional repressor domains (KRAB, DNMT3A or DNMT3L) did not [82]. This system was also applied in a mouse model of heritable eye disease (retinitis pigmentosa), where AAV vector-based delivery of dCas9-KRAB induced gene repression, which prevented the degradation of photoreceptors and improved visual function [82]. The transcriptional repressor dCas9-KRAB was packaged into lentiviral vectors and infused into the mouse brain to perform multiplex gene silencing [83]. In another study, KRAB was fused to the smaller dSaCas9, which is compatible with AAV vector-based delivery, and induced gene silencing in vivo, which was associated with reduced cholesterol levels [81]. The gene silencing was still observed after 24 weeks, which indicates the long-term potential of using CRISPR/Cas9 derivatives in adult mice [81].

The dCas9-KRAB, but also dCas9 fused to the epigenetic repressor LSD1 (lysine-specific demethylase 1) are electroporated in the chick embryo to inactivate enhancers [80]. dCas9-VP64 in combination with the SAM system (MS2 protein-fused additional VP-64 domains) was used as well in the chick embryo to activate gene expression [80]. dCas9 can also be fused to an engineered prokaryotic DNA methyltransferase MQ1 to achieve locus-specific methylation in mice via zygote microinjection [84]. This indicates its potential use in early development [84]. Interestingly, the increased methylation is still observed after birth, indicating its heritability for at least three weeks [84].

Altogether, this shows the in vivo applications of different CRISPR/Cas derivatives (summarized in Table 4). Both transcriptional activation and repression are achieved in different species such as drosophila and mice, in both the embryonic and adult state, which can induce alterations in phenotype in vivo. This can be used to study gene function and the associated phenotype in disease settings in vivo, and aid unraveling novel pathology-contributing genes as well as potential novel therapies.

## 6. Effective Gene Delivery In Vivo

Safety and effectiveness are the two major concerns for delivering dCas9 derivatives into the affected cells, and thus influence treatment efficacy. Basically, three different formats can be used as delivery methods: gene expression plasmids, viral vectors and ribonuclear complexes. The selection of the delivery system ideally should provide: (1) tissue-specificity, (2) target cell entry ability and (3) without/low immunogenicity. Initially, as proof-of-concept, many of the therapeutic applications of the dCas9 derivatives were performed on animal models via the electroporation of plasmid DNA [42,76]. Electroporation can be highly toxic since it can harm the cell membrane. In some cases, this leads to permanent permeabilization of the membrane [85]. Viral vector-based gene delivery systems have been widely used in gene therapy and even entered clinical trials in some cases [86]. The most widely used are lentiviral and AAV vectors, each one having its own advantages and disadvantages. For example, a lentiviral vector has a large packaging capacity up to 8.5 kB, long-lasting transgene expression and is capable of transducing non-dividing cells [87]. However, a lentiviral vector is oncogenic, immunogenic and is likely to make a transgene insertion into the host genome, which largely constrains its applications [87]. On the other hand, AAV vectors have a large variety of target tissues with low immunogenicity and non-oncogenicity [88]. AAV vectors have a very small genome size however, and therefore a low packaging capacity, which makes it unable to deliver a large transgene within a virus [88]. Instead of delivering DNA into the cells, the dCas9 derivatives can also be first transcribed into mRNA and delivered into cells via ribonucleoproteins. The transient nature of this approach favors controlling the gene expression level and minimizes the window of immune activation [87]. Moreover, from a pharmaceutical point of view, ribonucleoproteins are much easier to scale up for clinical use and can be chemically functionalized with ligands to obtain target cell specificity [87].

## 7. Conclusions

The discovery of the CRISPR/Cas9 system has revolutionized the field of epigenetic and genetic editing in the pre-clinic. This is a fast-growing field where different Cas9 proteins have been discovered with each their distinct features (e.g., length and recognition sites). The discovery of endonuclease-deficient Cas9 has led to the development of different CRISPR/dCas9 derivatives, which can be used to perform distinct functions such as tracking, transcriptional activation and repression, and base editing. Some of these dCas9-based derivatives have also been applied in vivo, where they contribute to transcriptional activation or repression which can lead to changes in disease phenotypes. This holds great promise for genetic screens to identify novel genes which are associated with a disease phenotype, and of course for potential therapies. Even though the discovery of high-fidelity Cas9 has significantly improved off-target effects, future research needs to be directed to improve methods to detect off-target effects (e.g., a recent study has demonstrated that engineering a hairpin secondary structure onto the spacer region of single-guided RNAs (hp-sgRNAs) can increase the specificity by several orders of magnitude) [89]. In addition, recent advancements in the unbiased detection of CRISPR off-target effects (DISCOVER-Seq) in patient-derived induced pluripotent stem cells directs us towards in situ off-target discovery within individual patient genotypes during therapeutic genome modulation [90]. Furthermore, future research needs to be directed at optimizing delivery methods for the clinical use of CRISPR/Cas (e.g., a recent study showed proof of concept of a self-deleting AAV-CRISPR system) [91]. Altogether, the CRISPR/Cas-based epigenetic and genetic editing is a fast-growing field with opportunities to develop novel applications altogether and increase the scale of already existing CRISPR/Cas-based applications.

## Figures and Tables

**Table 1:** Comparison of gene editing techniques.

	Meganucleases	ZFN	TALEN	CRISPR
Flexible localization	Complex	Limited	Average	Almost total
Nuclease construction	Laborious	Significant	Significant	Simple
In vitro testing	Laborious	Significant	Significant	Simple
Targeting efficiency	Not reported	Limiting factor	Average	Good
Off-target effects	Low	Moderate	Moderate	High
Multiplexing	No	No	No	Yes
Time investment	Very high	High	Moderate	Low
Cost	Very high	High	Average	Low

Colors indicate different scores: green (high), blue (middle) and peachpuff (low).

**Table 2:** Comparison of Cas proteins.

Nuclease	Name	Protein Size(aa)	WT/Mutants	PAM (5′-3′)	Protospacer Length	Target/Type of DSB	Pros/Cons	In Vivo/In Vitro
Cas9 (HNH, RuvC)	SpCas9	1,368aa	WT	NGG	20nt	DNA/Blunt end	Most commonly used/Large protein size	in vitro, in vivo [11]
			VQR	NGAN			Different PAM specificities/Large protein size	in vitro, in vivo [15]
			EQR	NGAG				in vitro, in vivo [15]
			VRER	NGCG				in vitro, in vivo [15]
	SaCas9	1,053aa	WT	NNGRRT			Small protein size/Relatively strict PAM	in vitro, in vivo [19]
			KKH	NNNRRT				in vitro, in vivo [20]
	FnCas9	1,629aa	WT	NGG			Less restrictive PAM/Large protein size, less application examples	in vitro, in vivo [21]
			RHA	YG				In vitro [21]
	NmCas9	1,082aa	WT	NNNNGATT	24nt		Small protein size/Strict PAM	in vitro, in vivo [17]
	St1Cas9	1,121aa	WT	NNAGAAW	20nt		Small protein size/Strict PAM	in vitro [16]
	BlatCas9	1,092aa	WT	NNNNCNDD	21nt		Less restrictive PAM, small protein size/Less application examples	In vitro [18]
Cas12 (RuvC-like)	AsCas12a/Cpf1	1,307aa	WT	TTTN	23nt	DNA/Staggered end	Various unique characteristics/restrict PAM, with 5′ overhangs	in vitro, in vivo [22]
	LbCas12a/Cpf1	1,228aa	WT	TTTN				in vitro, in vivo [22]
	BhCas12b	1,140aa	WT	ATTN	23nt	DNA/Staggered end	High specificity	in vitro [23]
	Cas12c	1253aa	WT	TG/TN	n.a.	DNA	Less restrictive PAM, small protein size	in vitro[24]
	Cas12g	768aa	WT	Not required	24nt			
	Cas12h	871aa	WT	RTR	n.a.			
	Cas12i	1055aa	WT	TTN	28nt			
	CasX/Cas12e	987aa	WT	TTCN	20nt	Staggered end	Very Small protein size	in vitro[25]
Cas14 (RuvC)	Cas14a	529aa	WT	Not required	25nt	DNA	Very small protein size, target ssDNA	in vitro [32]
Cas13 (2xHEPN)	LshCas13a	1427aa	WT	3′ A, U, or C (not required by all orthologs)	28nt	RNA	Very flexible PFS, target RNA	in vitro [27]
	LwaCas13a	1152aa	WT		28nt			in vitro [26]
	PspCas13b	1124aa	WT		30nt			in vitro [26]
	RfxCas13d	979aa	WT		30nt		Very small protein size, target RNA	in vitro [29]

Colors indicate different Cas protein families. A: Adenine; C: Cytosine; G: Guanine; T: Thymine; D: A or G or T; R: A or G; W: A or T; Y: C or T; N: any base; n.a.: not applicable.

**Table 3:** dCas protein derived gene modulators. Colors indicate different scores.

	Effector Domains	Function	Purpose	Reference
dCas9	GFP/BFP	Gene visualization	Tracking	[33]
	SunTag (10xGCN4)	Adaptor domain	Recruitment of other effector domains	[34]
	APOBEC	“C” to “T” substitution	Base editing	[53,60]
	AID	“C” to “T/G” substitution	Base editing	[62,63]
	ABE	“A” to “G” substitution	Base editing	[64]
	Reverse transcriptase	Reverse transcription	Base editing	[66]
	VP64	Transcriptional activation	Activation	[34,36,37]
	P65AD	Transcriptional activation	Activation	[36]
	VPR	Transcriptional activation	Activation	[38]
	p300	Histone acetylation	Activation	[40]
	TETs	DNA demethylation	Activation	[41,42,43,44]
	PRDM9	Histone methylation	Activation	[46]
	DOT1L	Histone methylation	Activation	[46]
	SMYD3	Histone methylation	Activation	[45]
	KRAB	Chromatin remodeling	Repression	[36,48,51]
	LSD1/KDM1A	Histone demethylation	Repression	[51]
	DNMTs	DNA methylation	Repression	[48,49,50]
	EZH2	Histone methyltransferase	Repression	[52]
	HDAC3	Histone deacetylation	Repression	[53]
dCas13	GFP	RNA visulization	Tracking	[26]
	ADAR	“A” to “I” substitution	Base editing (REPAIR)	[28,65,68,69,72]
	YTHDF1	Promote mRNA translation	Activation	[71]
	YTHDF2	Promote mRNA decay	Repression	[71]
	KRAB	Transcription repression	Repression	[26]

**Table 4:** In vivo applications of dCas9 derivatives.

Module	Species	Delivery Method	Feature	Reference
dCas9-TET1CD	Mouse	In utero electroporation/ Lentiviral vectors	Demethylation in brain of mouse fetuses/ demethylation in skin and brain	[42,79]
dCas9-TET3CD	Mouse	Lentiviral vectors	Gene re-activation and amelioration of kidney fibrosis	[44]
dSaCas9-KRAB	Mouse/Chicken	AAV8 vectors/ Electroporation	Gene silencing and lowering of cholesterol levels/ Inactivation of enhancers in the chick embryo	[80,81]
dCas9-LSD1/VP64	Chicken	Electroporation	Inactivation of enhancers in the embryo	[80]
dCas9-MQ1	Mouse	Zygote microinjection	Methylation in zygote	[84]
dCas9-10xGCN4 with p65-HSF1-SAM	Mouse	AAV8 vectors	Simultaneous transcriptional activation of multiple genes	[78]
dCas9-VPR	Drosophila	Cross breeding transgenic lines	Gene activation	[73,74]
dCas9-SunTag(VP64)	Mouse	AAV vectors	Gene activation/growth and tumorigenesis assay	[77]
dCas9 with MS2-p65- HSF1	Mouse	Electroporation	Light-mediated gene activation in muscle	[76]
dCas9-VP64	Mouse	Tail-vein injection of transgenic B-ALL cells	Gene activation/repression in cancer	[56]
dCas9-KRAB	Mouse	AAV vectors/Lentiviral vectors	Split-intein-mediated gene repression in retinitis pigmentosa/multiplex gene silencing in the brain	[82,83]
dCas9-VP64/MS2-p65-HSF1	Mouse	AAV vectors	Multiplexed activation of endogenous genes	[54]
dCas9-VPR	Rat	Lentiviral vectors	Increased protein levels of a target gene in diverse brain structures	[75]
